# Fabrication and Sensing Characterization of Ionic Polymer-Metal Composite Sensors for Human Motion Monitoring

**DOI:** 10.3390/s26020394

**Published:** 2026-01-07

**Authors:** Guoxiao Yin, Chengbo Tian, Qinghua Jiang, Gengying Wang, Leqi Shao, Qinglin Li, Yang Li, Min Yu

**Affiliations:** 1 Jiangsu Key Laboratory of Bionic Materials and Equipment, College of Mechanical and Electrical Engineering, Nanjing University of Aeronautics and Astronautics, Nanjing 210016, China; yinguoxiao@nuaa.edu.cn (G.Y.); tiancb@nuaa.edu.cn (C.T.); jiangqinghua@nuaa.edu.cn (Q.J.); wgying@nuaa.edu.cn (G.W.); slq@nuaa.edu.cn (L.S.); liqinglin@nuaa.edu.cn (Q.L.); yangli@nuaa.edu.cn (Y.L.); 2 State Key Laboratory of Mechanics and Control for Aerospace Structures, Nanjing University of Aeronautics and Astronautics, Nanjing 210016, China

**Keywords:** IPMC, sensor, sensing voltage, pressure, human motion monitoring

## Abstract

This work presents the fabrication and a systematic evaluation of an ionic polymer-metal composite (IPMC) sensor, focusing on its potential for human motion monitoring and human–computer interaction. The sensor was fabricated via a solution casting and electroless plating process, and its morphology characterized using scanning electron microscopy. The sensing performance was comprehensively assessed, revealing high sensitivity (1.059 mV/N) in the low-pressure region, a fast response time (~50 ms), and reliable stability over prolonged cyclic testing. Furthermore, the sensor can respond to both the magnitude and rate of applied mechanical stimuli. To explore its application potential, the IPMC was tested in scenarios ranging from input pattern recognition—including distinguishing mouse-click patterns, handwritten letters, and binary-encoded presses—to human motion monitoring, where it effectively captured and differentiated signals from facial expressions, swallowing, breathing, and joint movements. The results suggest that the developed IPMC sensor is a promising candidate for applications in wearable health monitoring and flexible interactive systems.

## 1. Introduction

Ionic polymer-metal composite (IPMC) is a typical electroactive polymer [1,2,3], which has the key characteristics of flexibility, light weight, simple structure, noiselessness, and good biocompatibility [4,5,6]. Its driving performance has been widely studied in applications, such as bionic fish [7,8,9,10,11], flexible grippers [12,13,14], medical catheters [15,16,17], bionic flowers [18], micropumps [19,20], bionic robots [21,22,23,24], and others [25,26]. Furthermore, it has attracted increasing research interest in recent years as a sensor [27] and is widely used to measure displacement, velocity, and pressure [28,29,30]. Its suitability for operation in liquid [31,32,33] or humid environments [34,35] further broadens its potential sensing applications. For example, Park et al. [36] utilized the sensitivity of IPMC to curvature changes by attaching it to the biceps brachii to monitor muscle state variations during elbow flexion and extension. Meanwhile, Lee et al. [37] proposed an AI-assisted laryngeal sensor that is safe, portable, and non-invasive for detecting the swallowing process in patients.

Structurally, IPMC typically consists of a polymer ion-exchange membrane (e.g., Nafion, Aquivion, or SPEEK) sandwiched between metal electrodes (e.g., Pt, Au, Pd) [38,39]. Its sensing mechanism originates from the redistribution of mobile ions within the polymer matrix under external mechanical strain, which generates a corresponding electric potential across the electrodes [39,40]. Research on IPMC pressure sensing has progressed in exploring factors such as contact area [41], structural design [42], and operational mode [43]. For instance, studies have shown that sensitivity can be significantly enhanced through shape optimization or by utilizing shear mode. However, existing work has predominantly focused on sensitivity metrics, while systematic evaluations of other critical parameters for practical applications, such as response time and long-term stability, have received less attention.

Therefore, to enable its application in dynamic scenarios such as human motion monitoring, a more comprehensive characterization of IPMC’s pressure-sensing performance is necessary. This work aims to address this gap by systematically evaluating the sensitivity, response time, and stability of fabricated IPMC pressure sensors using a custom-built experimental platform. Furthermore, we demonstrate their functionality in several application-oriented tests. This study provides a foundational assessment of IPMC sensors for potential use in wearable electronics and human–computer interaction systems.

## 2. Materials and Methods

### 2.1. Preparation of IPMC Samples

#### 2.1.1. Nafion Membrane Preparation and Treatment

**Figure 1:**
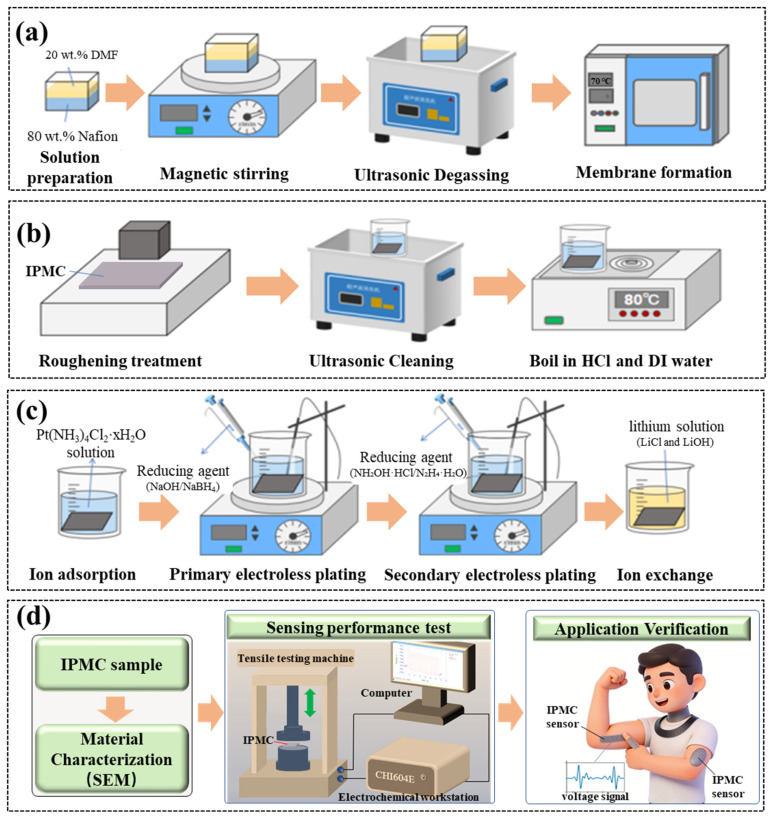
Preparation process diagram of IPMC: (**a**) preparation of Nafion substrate membrane by solution casting; (**b**) roughening treatment of Nafion substrate membrane; (**c**) fabrication of IPMC by electroless plating; (**d**) experimental flow chart.

A specified volume of Nafion solution was poured into a homemade rectangular silicone mold (with dimensions of 50 mm in length and 25 mm in width). Subsequently, a volume of dimethylformamide (DMF) solution equal to one-quarter of the Nafion solution volume was added. The silicone mold containing the Nafion solution was then placed on a magnetic stirrer, and a magnetic stir bar was introduced to mix the Nafion and DMF solutions uniformly for 6 h. Following this, the mold containing the homogenized mixture was transferred to an ultrasonic cleaner and subjected to sonication for 30 min to remove air bubbles from the solution. Finally, the mold with the solution was placed in a vacuum drying oven at 70 °C for 72 h to allow the solution to solidify into a membrane. The preparation procedure is illustrated in Figure 1a.

#### 2.1.2. Nafion Membrane Treatment

To facilitate the deposition of platinum particles, the dried Nafion membrane was roughened with an abrasive for 30 min to increase its surface roughness. Subsequently, it is cleaned with deionized water and sonicated for 30 min to remove surface impurities. Finally, it is acid-boiled with dilute hydrochloric acid at 80 °C for 30 min, followed by boiling in water at 80 °C for 30 min, as shown in Figure 1b.

#### 2.1.3. Chemical Plating Preparation of IPMC

The preparation process of IPMC mainly includes ion adsorption, primary chemical plating, secondary chemical plating and ion exchange, as shown in Figure 1c. The specific preparation process is described below.

(1) Ion adsorption: The pretreated Nafion membrane was immersed in a platinum-ammine complex solution (prepared from [Pt(NH_3_)_4_]Cl_2_, ammonia, and deionized water) for 12 h at room temperature in the dark, allowing Pt(NH_3_)_4_^2+^ ions to diffuse into the membrane.
(2) Primary electroless plating: After ion adsorption, the Nafion membrane was immersed in deionized water and placed on a magnetic heating stirrer (KEMS-DSS, Nanjing Kohl Instrument Co., Ltd., Nanjing, China). Adjust the temperature to 42 °C and the speed to 100 r/min. A reducing agent was added every 30 min, consisting of a mixture of 1.5% NaBH_4_ and 0.5% NaOH, and the temperature was increased by 3 °C every half hour, rising from 42 °C to 62 °C. After the plating was completed, the sample was immersed in 2% dilute hydrochloric acid overnight.
(3) Secondary electroless plating: In order to form denser and more uniform platinum electrodes on the surface of Nafion membrane, it is necessary to carry out secondary chemical plating. Before the secondary plating, the base membrane after primary plating was boiled twice to remove any residual acid on the surface. Prepare platinum ammonia solution and secondary plating reducing agents (NH_2_OH·HCl and N_2_H_4_·1.5H_2_O). A reducing agent was added every 30 min until the reaction was complete. Finally, the IPMC was cleaned and boiled after secondary plating and stored in dilute hydrochloric acid.
(4) Ion exchange: Since different types of cations have a significant impact on the sensing performance of IPMCs, and the use of Li^+^ as the internal cation helps enhance their sensing performance [44,45], the prepared IPMC samples were immersed in a prepared lithium solution for 48 h to complete the ion exchange process.

#### 2.1.4. Sample Preparation and Cutting

To obtain test samples with consistent geometry, the sheet-IPMC was cut into rectangular strips using a sharp scalpel. All samples used for sensing characterization, unless otherwise specified, were fabricated to identical dimensions of 7.0 mm in length, 7.0 mm in width, and 0.45 mm in thickness. This ensures that the comparisons of sensing performance are based on samples with uniform geometric parameters.

### 2.2. Experimental Tests and Setup

#### 2.2.1. Characterization

The surface and cross-sectional morphologies of the IPMC samples were characterized using a scanning electron microscope (SEM) (Quattro S, Thermo Fisher Scientific, Waltham, MA, USA). The cross-sections of the IPMC samples were obtained via cryogenic brittle fracture in liquid nitrogen, and all test samples were subjected to gold sputtering treatment prior to SEM observations.

#### 2.2.2. Water Uptake Test

A precision balance (AL204, Mettler Toledo, Greifensee, Switzerland) was used to measure the mass (denoted as *m*) of the IPMC sample. Under room temperature conditions, the sample mass was measured at 2 min intervals. The water uptake of the IPMC at different air exposure times was subsequently calculated using Equation (1).

(1) $$\mathrm{Water}\;\mathrm{uptake}\left(\%\right)=\frac{m-m_{\mathrm{dry}}}{m_{\mathrm{dry}}}\times 100$$

where *m* is mass of the IPMC specimen, *m*_dry_ is the dry mass of the IPMC sample.

#### 2.2.3. Sensing Performance Tests

The open-circuit voltage measured using an electrochemical workstation (CHI604E, Shanghai Chenhua Instrument Co., Ltd., Shanghai, China) was adopted as its sensing voltage. To more accurately characterize the sensing voltage of the IPMC, relative voltage values were used for description in this experiment. A universal tensile testing machine (ZQ-950, Dongguan Zhiqu Precision Instruments Co., Ltd., Dongguan, China) was employed to apply the loading force and control the loading speed. To ensure the reliability of the test results, each sample was tested at least three times. Prior to all sensing performance tests, the IPMC samples were equilibrated under ambient laboratory conditions (~25 °C, 40–60% RH) for over 1 h to ensure a stable hydration state.

### 2.3. Overview of Experimental Workflow

The experimental workflow comprised three main phases: (1) material characterization (e.g., SEM), (2) systematic evaluation of fundamental sensing properties (sensitivity, response time, stability), and (3) validation of application potential through pattern recognition and human motion monitoring tests. This overall process is summarized in Figure 1d.

## 3. Results and Discussion

### 3.1. SEM Results

**Figure 2:**
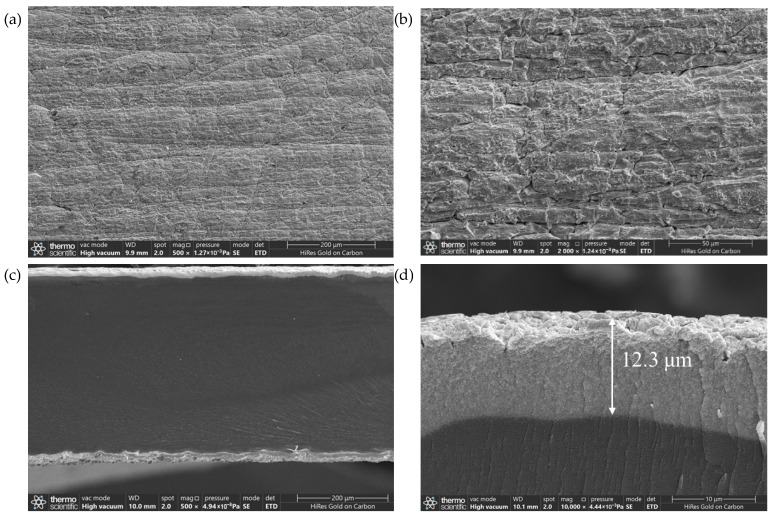
SEM results of IPMC: (**a**) surface SEM image (×500 magnification); (**b**) surface SEM image (×2000 magnification); (**c**) Cross-sectional SEM image; (**d**) electrode thickness.

Figure 2a shows the surface SEM image of the IPMC, where distinct grinding marks and micro-scale cracks are distributed across the surface. This roughened morphology is more clearly revealed in the higher-magnification view of Figure 2b. Such a roughened surface helps to increase the contact area between the Pt electrode and the Nafion membrane, enhancing the bonding strength between them, thereby improving the service life of the IPMC electrode and contributing to the formation of a favorable metal-polymer interface layer. The cross-sectional SEM image in Figure 2c reveals that the IPMC exhibits a typical sandwich structure: the upper and lower layers are Pt metal electrodes (with an electrode thickness of 12.3 μm as seen in Figure 2d), and the middle layer is a dense Nafion membrane. The electrode layers are tightly bonded to the polymer electrolyte with a clear interface, indicating good adhesion between the electrode and the substrate membrane during the fabrication process, without noticeable delamination or void defects. Sufficient electrode thickness forms the basis for a low-resistance, high-quality metal/polymer interface. Furthermore, this dense and uniform electrode/electrolyte interface layer is crucial for enhancing the overall sensing performance of the IPMC sensor [29].

### 3.2. Water Uptake

Considering that variations in the water uptake of IPMC affect its sensing voltage [46], in order to mitigate this influence, the variation in water uptake of IPMCs with air exposure time was investigated to identify a relatively stable water uptake, thereby reducing the impact of water uptake changes on the sensing voltage.

**Figure 3:**
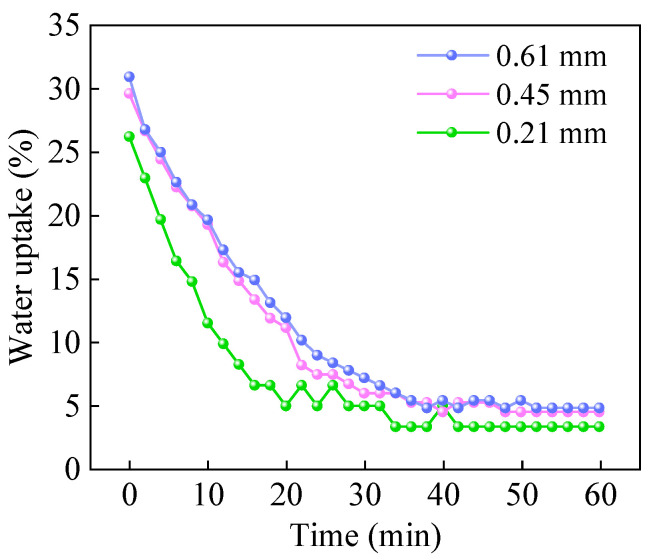
Variation in IPMC water uptake with air exposure time.

As shown in Figure 3, under ambient conditions, the initial water uptakes of the three IPMC samples with thicknesses of 0.21 mm, 0.45 mm, and 0.61 mm were 26.23%, 29.63%, and 30.95%, respectively. After air exposure for 42 min, 48 min, and 52 min, the water uptakes stabilize at 3.28%, 4.44%, and 4.79%, respectively. The water uptake exhibits an exponential decay trend over time: upon air exposure, the water uptake drops rapidly, then the rate of decline gradually slows, and eventually stabilizes. This behavior corresponds to the diffusion and evaporation of moisture from the interior of the material to the dry ambient air. Thicker IPMC samples show higher initial water uptake, indicating that thicker materials can absorb and retain more water. Furthermore, thicker samples require a longer time to reach a stable water uptake and demonstrate better water-retention capability, which is attributed to the longer diffusion path required for moisture to travel from the interior to the surface. In summary, to ensure that subsequent sensing performance tests are conducted under stable water-content conditions, all IPMC samples must be preconditioned via air exposure for a sufficient duration (60 min) before testing, in order to eliminate the interference caused by variations in water uptake on the sensing voltage signal output.

### 3.3. Sensing Performance

The sensing characteristics of a sensor are the external manifestation of the intrinsic relationships among its structural parameters. To understand the sensing properties of the IPMC, this section investigates its sensitivity, response time, and stability.

#### 3.3.1. Sensitivity

The sensitivity (*k*) was calculated as the ratio of the change in sensing voltage (Δ*V*) to the change in applied force (Δ*F*):

(2) $$k=\frac{\Delta V}{\Delta F}=\frac{V-V_{0}}{F-F_{0}}$$

where *k* is the sensitivity; *V* is the sensing voltage of the IPMC sensor; *V*_0_ is the initial sensing voltage of the IPMC sensor; *F* is the force applied to the IPMC sensor; *F*_0_ is the initial force applied to the IPMC sensor.

**Figure 4:**
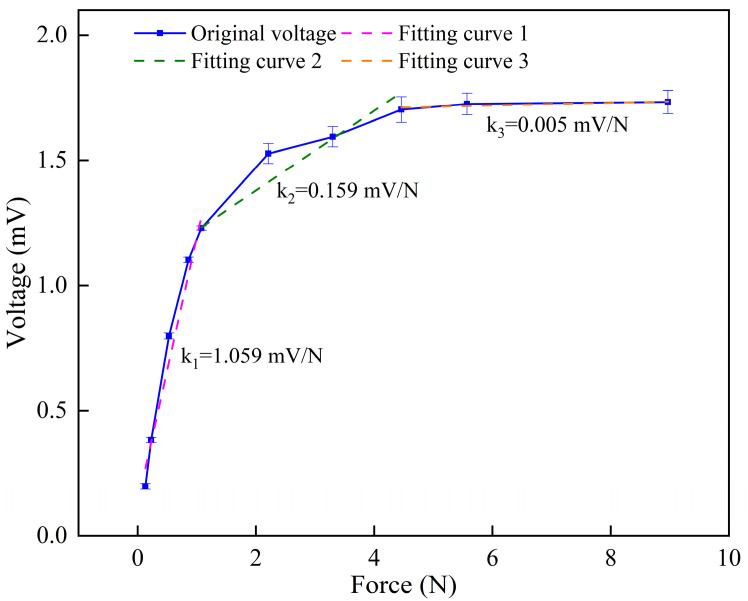
Sensitivity of the IPMC sensor.

As shown in Figure 4, the sensing performance curve of the IPMC sensor can be divided into three distinct regions: S1 (0~1.08 N), S2 (1.08~4.46 N), and S3 (4.46~8.97 N). The corresponding sensitivities were *k*_1_ = 1.059 mV/N, *k*_2_ = 0.159 mV/N, and *k*_3_ = 0.005 mV/N, respectively. Evidently, the sensitivity of the IPMC pressure sensor in the low-pressure range (Region S1) is significantly higher than that in the medium- and high-pressure ranges (Regions S2 and S3). This characteristic indicates that the IPMC sensor is particularly suitable for micro-force measurement in practical applications.

#### 3.3.2. Stability

**Figure 5:**
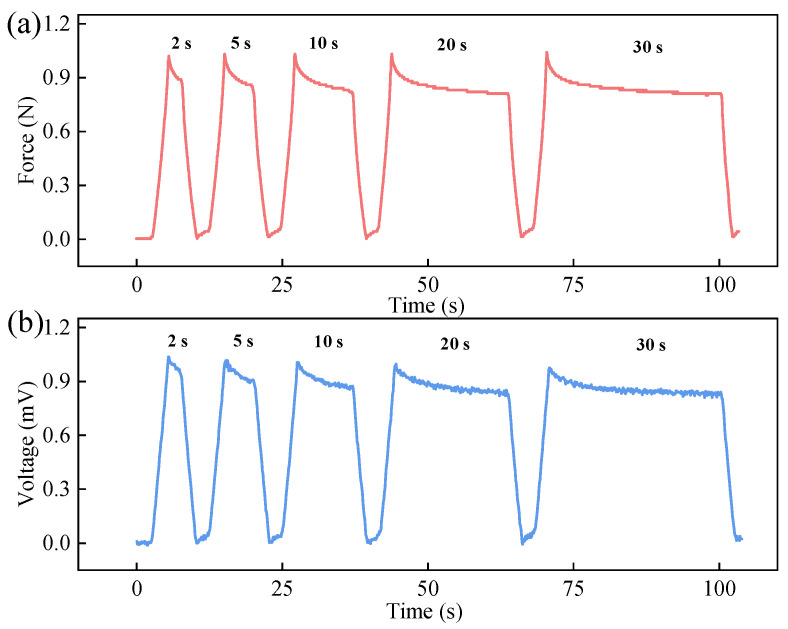
Response of IPMC sensor to steady-state forces: (**a**) loading force curve; (**b**) sensing voltage curve.

The tensile testing machine was used to apply fixed pressures with varying holding times (2 s, 5 s, 10 s, 20 s, and 30 s) to the IPMC at a constant loading speed, characterizing its sensing voltage response under static loading. The results are presented in Figure 5.

Figure 5a shows the applied force curve, and Figure 5b displays the corresponding sensing voltage. As observed, upon initial contact, a transient spike in both the applied pressure and the IPMC’s sensing voltage occurs due to the high contact stress. Subsequently, both signals stabilize as the contact settles. Crucially, the voltage curve exhibits high temporal synchronization with the force curve. Every step change in the loading force triggers an immediate step response in the sensing voltage without noticeable delay (Figure 5b).

Across all tested holding durations, the IPMC sensor consistently generated stable and synchronized voltage responses. These results demonstrate that the IPMC sensor possesses excellent responsiveness and stability for static force measurement.

#### 3.3.3. Synchronization

**Figure 6:**
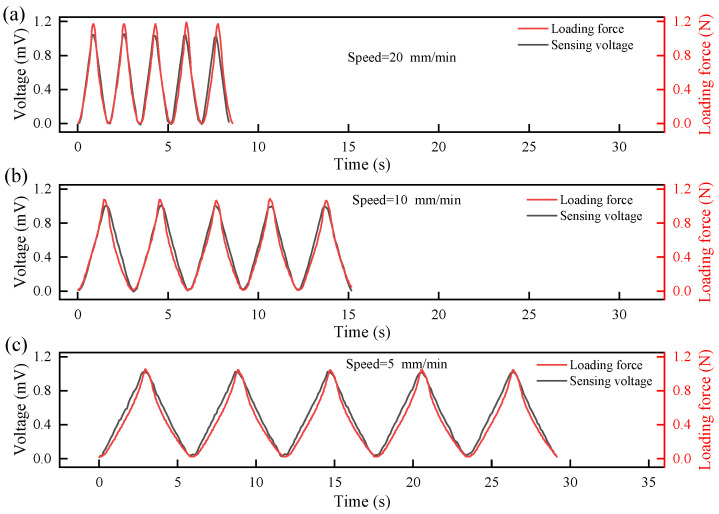
The actual variation curve of sensing voltage of IPMC with applied loading force: (**a**) loading speed = 20 mm/min; (**b**) loading speed = 10 mm/min; (**c**) loading speed = 5 mm/min.

To assess the real-time tracking capability, the output voltage was compared with the dynamically applied loading force at rates of 5, 10, and 20 mm/min. The results are shown in Figure 6. As can be seen from Figure 6, under all three loading speeds, the IPMC generates corresponding sensing voltages in response to pressure changes, and both the sensing voltage curve and the loading force curve exhibit a sinusoidal-like variation. The shapes of the loading force and the sensing voltage are highly similar, and the repeated test curves show good overlap, indicating excellent morphological synchronization. The strong synchronization of the IPMC material ensures consistency and reliability in its output signal, which facilitates relatively accurate and stable dynamic force sensing and decoupling in practical applications.

#### 3.3.4. Response Time

**Figure 7:**
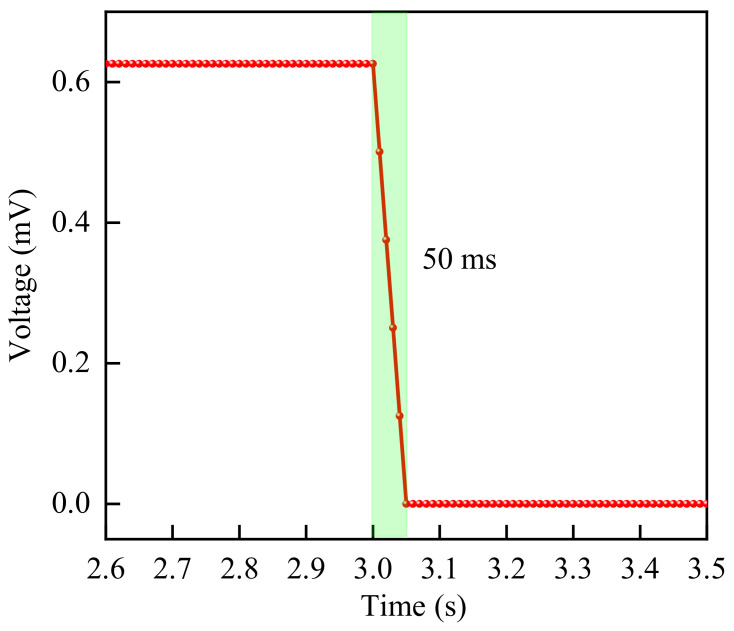
Sensing voltage variation curve of IPMC after pressure unloading.

Response time refers to the time required for a sensor to react to an external stimulus, i.e., the time needed for the sensor to convert an input excitation signal into a stable output electrical signal. Response time is also one of the important indicators characterizing the sensing performance of IPMC. Therefore, this section tested the response time of IPMC, and the test results are shown in Figure 7. Figure 7 shows the change in sensing voltage after the loading force applied to the IPMC is rapidly released. As can be seen from Figure 7, when the pressure on the IPMC is quickly removed, the sensing voltage of the IPMC can rapidly return to its initial state, with a response time of approximately 50 ms. This indicates that the IPMC material, as a sensor, possesses fast response characteristics and is suitable for dynamic measurement environments.

#### 3.3.5. The Influence of Loading Force and Loading Speed

**Figure 8:**
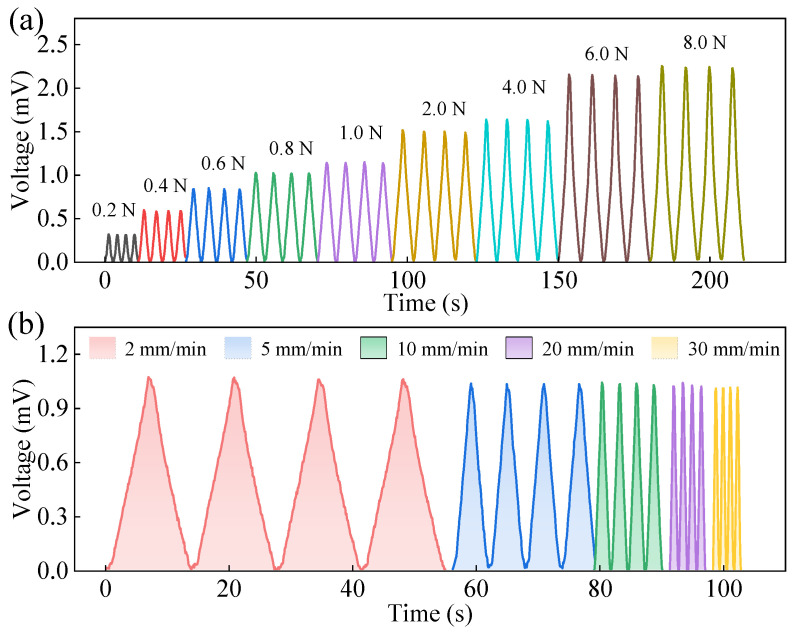
Sensing voltage of IPMC as a function of loading force and loading speed: (**a**) sensing voltage under different loading forces; (**b**) sensing voltage under different loading speeds.

This section investigates the sensing voltage response of the IPMC sensing material under different loading forces and loading speeds to further evaluate its performance in practical applications. For the pressure tests, the force applied by the universal tensile testing machine was set from 0.2 N to 8 N. For the loading speed tests, a constant force of 1 N was maintained while the loading speed was varied at 2 mm/min, 5 mm/min, 10 mm/min, 20 mm/min, and 30 mm/min. Each parameter was tested for four repeated cycles. The corresponding results are presented in Figure 8.

Figure 8a and Figure 8b, respectively, show the sensing voltage changes in the IPMC under different loading forces and loading speeds. As seen in Figure 8a, the sensing voltage of the IPMC gradually increases with higher applied loading force. When the loading force rises from 0.2 N to 8.0 N, the corresponding sensing voltage increases from 0.32 mV to 2.24 mV, indicating a very noticeable enhancement.

From Figure 8b, it can be observed that the variation in loading speed does not significantly affect the peak value of the sensing voltage. For example, at loading speeds of 2 mm/min, 5 mm/min, 10 mm/min, 20 mm/min, and 30 mm/min, the corresponding sensing voltages are approximately 1.06 mV, 1.03 mV, 1.03 mV, 1.02 mV, and 1.01 mV, respectively. Faster loading speeds lead to a steeper rise in the sensing voltage signal and a narrower peak, whereas slower loading speeds produce a broader and more gradual voltage signal.

Moreover, the voltage curves (both waveform and peak values) from the four repeated tests exhibit good consistency, preliminarily indicating that the IPMC has good response consistency and test repeatability under the given conditions. In summary, the prepared IPMC sensor can not only sense the “magnitude of force” but also distinguish the “rate of force change”, demonstrating its capability for dynamic multi-parameter pressure sensing.

#### 3.3.6. Repeatability

**Figure 9:**
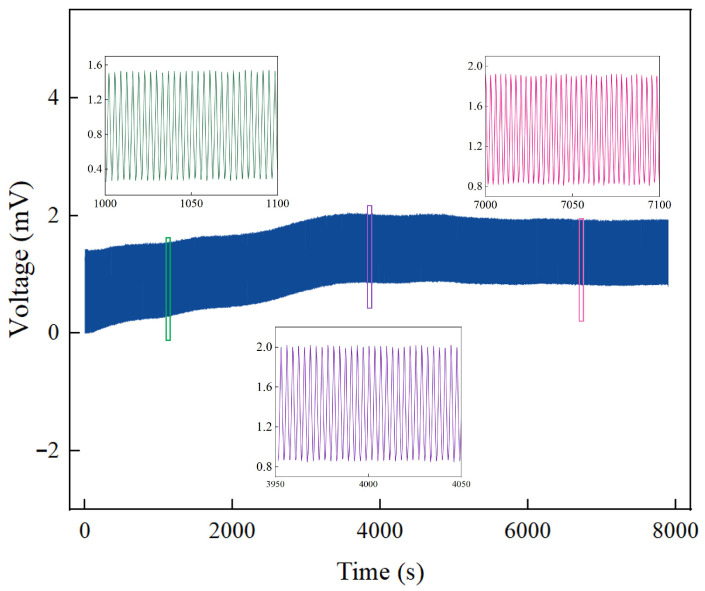
Cycle test of the IPMC sensor.

To verify the long-term reliability of the IPMC sensing material, cyclic tests were conducted over 8000 s (approximately 2200 cycles) using a tensile testing machine. The results are shown in Figure 9.

As observed in Figure 9, the IPMC sensor generated consistent and repeatable voltage signals throughout the prolonged loading–unloading test. Although a gradual decrease in the signal amplitude was observed under stable ambient laboratory conditions (~25 °C, 40–60% RH)—from approximately 1.23 mV at 1000 s to about 1.15 mV at 4000 s and 1.09 mV at 7000 s, representing an overall attenuation of 11.3%—the waveform itself remained well-defined without significant distortion or failure. This indicates that the sensor maintains good signal stability and repeatability over extended cyclic operation within this specific environmental range.

In summary, the IPMC material prepared in this work demonstrates promising potential for practical applications requiring long-term periodic monitoring, and also lays the groundwork for subsequent tests in human motion behavior monitoring.

### 3.4. Application Study

Based on the experimental results demonstrating the favorable sensing performance of the IPMC sensing material prepared in this study, this section further validates its application potential through input pattern recognition tests and human motion monitoring tests. All application tests were performed using an electrochemical workstation for sensing voltage signal acquisition, with the sampling rate set at 100 Hz. Prior to the formal tests, the sensors were kept stationary for 1 h in a laboratory environment at room temperature (~25 °C, 40–60% RH) to ensure the stable sensing performance of the IPMC materials.

#### 3.4.1. Input Pattern Recognition Tests

**Figure 10:**
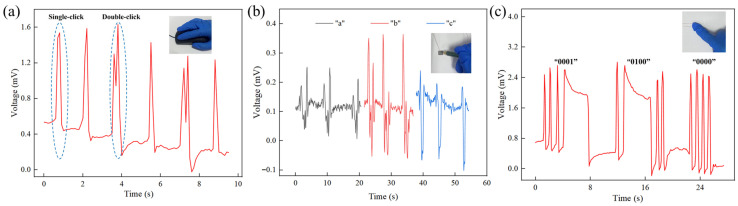
IPMC sensor application tests: (**a**) recognition of mouse click frequency; (**b**) English letter recognition test; (**c**) binary information transmission test.

Mouse Click Pattern Recognition test: To leverage its flexibility and high sensitivity for human–computer interaction, the IPMC sensor was attached to the tester’s right index finger. In the mouse click pattern recognition test experiment, the IPMC sensing material was attached to the index finger of the tester’s right hand. The tester performed consecutive single- and double-click actions on a mouse. As shown in Figure 10a, a single click produced a voltage signal with one peak, while a double click yielded two distinct peaks. This clear distinction enables reliable recognition of basic click patterns, highlighting the sensor’s potential for intuitive control of flexible/wearable devices.

English letters recognition: The IPMC’s sensitivity to pressure and trajectory variations was exploited for letter recognition. In this section, the IPMC sensing material was used as a handwriting pad, and the three letters “a”, “b”, and “c” are drawn on it with a pen to observe the corresponding voltage changes. As depicted in Figure 10b, when the three letters “a”, “b”, and “c” are drawn continuously, there are significant differences in the peak values, directions, and waveforms of the sensing voltages corresponding to the three letters. Based on these differences in sensing voltages, the corresponding letters can be identified. This result demonstrates the potential of flexible IPMC sensing materials in recognizing complex gesture patterns such as letter handwriting.

Information transfer test: To demonstrate encoded information transfer, Figure 10c presents the voltage response curve obtained during binary information transmission via finger presses on the IPMC sensor. In this experiment, a binary encoding scheme was adopted, where a brief press represents “0” and a sustained press represents “1”, enabling the transmission of any Arabic numeral from 0 to 9. The results show that the sensor responds promptly with stable signals, clearly distinguishing between the “0” and “1” states, thereby confirming its capability to transmit information in encoded form. This suggests potential applications in secure access or monitoring systems.

In summary, the IPMC sensing material has shown promising recognition capabilities across different interaction modes (mouse clicks/letter recognition/information transmission). It should be noted that these are proof-of-concept demonstrations under controlled, single-user conditions. Performance may vary with inter-user differences in writing style or pressure. A comprehensive evaluation involving multiple subjects will be an essential next step to establish statistical robustness and generalizability for practical applications.

#### 3.4.2. Human Motion Monitoring

**Figure 11:**
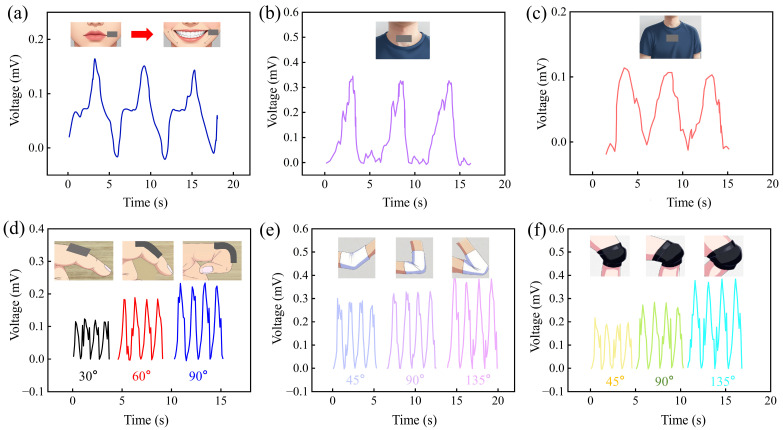
The electrical signal response of IPMC sensors under different human behaviors: (**a**) facial smile; (**b**) throat swallowing; (**c**) deep breathing in the chest; (**d**) finger joint bending; (**e**) elbow joint bending; (**f**) knee joint bending.

To further verify the application potential of the IPMC sensing material in human motion behavior analysis, the IPMC sensor was fixed to different test sites on the human body (face, throat, chest, finger joints, elbow joints, and knee joints) with medical tape. The signal acquisition was conducted in a laboratory environment at room temperature, and the test results are presented in Figure 11.

The IPMC sensing material was attached to the cheek using medical tape. When a smiling action occurs, IPMC sensing materials generate a corresponding sensing voltage. The voltage curve in Figure 11a indicates that the sensing voltages recorded during three consecutive smiles demonstrated good consistency. This shows that the IPMC sensing material is capable of recognizing smiling motions and can clearly capture subtle changes in facial expressions.

As shown in Figure 11b,c, the sensor detected distinct signals corresponding to swallowing and deep breathing, respectively. Both generated clear, cyclical responses. Notably, the voltage amplitude for swallowing was significantly higher than for breathing. This is primarily because the rapid, large-amplitude movement of the laryngeal cartilages during swallowing imposes a strong, abrupt dynamic strain (high strain rate and amplitude) on the sensor. In contrast, the gentle thoracic movement during deep breathing results in weaker, slower strain. Consequently, the swallowing signal appears as a sharp voltage pulse, while the breathing signal is smoother (Figure 11b,c). Based on the differences in peak voltage and waveform characteristics, the swallowing and deep breathing actions can be effectively distinguished.

Figure 11d–f illustrate the relationship between the bending angles of the finger joint, elbow joint, and knee joint and the response of the IPMC sensing material. As shown in the figures, the sensing voltage of the IPMC increases with the enlargement of the joint bending angle, primarily because a larger angle induces more significant mechanical deformation of the IPMC sensor, thereby driving more internal ions to undergo directional migration and generating a stronger induced potential difference. In addition, joints of different sizes, from small finger joints to large knee joints, exhibit consistent response patterns. This feature highlights the good adaptability of the sensor to mechanical stimuli of different scales, further verifying its enormous potential in the field of human joint motion monitoring, such as real-time tracking of rehabilitation training progress, evaluation of limb motion function, and development of wearable motion capture systems.

In summary, these findings confirm the application potential of IPMCs in both input pattern recognition and human motion analysis, establishing a solid foundation for the future implementation of advanced intelligent algorithms in embedded systems.

Although this study has preliminarily verified the feasibility, several challenges remain, including long-term signal drift, motion artifacts during strenuous exercise, and signal variations due to individual physiological differences. Future work will focus on developing encapsulation technologies for better stability in humid environments and integrating machine learning algorithms for real-time, multi-channel signal pattern recognition. The ultimate goal is to provide a novel, flexible sensing solution for personalized medical rehabilitation and intelligent human–machine interaction.

## 4. Conclusions

This study fabricated IPMC sensors with promising performance and conducted systematic characterization and application exploration. SEM analysis confirmed that the prepared IPMC possesses a uniform “sandwich” structure, dense electrode layers, and good electrode-substrate bonding, laying a structural foundation for stable sensing performance.

Regarding sensing performance, the test results demonstrate that: (1) the IPMC sensor exhibits high sensitivity (1.059 mV/N) in the low-pressure region (<1.08 N), making it particularly suitable for microforce detection; (2) it features fast response (~50 ms) and excellent signal synchronization, enabling real-time tracking of dynamic force changes; (3) long-term cyclic tests showed good signal consistency and stability with controllable attenuation, confirming its reliability for prolonged operation; (4) the sensor output responds to both the magnitude and the rate of applied pressure, reflecting multi-parameter sensing capability.

In terms of application validation, this research demonstrates the potential of IPMC sensors from two perspectives: active interaction and passive monitoring. For active interaction, the IPMC sensor successfully achieved accurate recognition of mouse-click patterns, handwritten letter trajectories, and binary-encoded information, highlighting its potential for developing novel flexible human–computer interfaces. For passive monitoring, the sensor was effectively used to capture various human physiological and motion signals, including facial smiling, throat swallowing, chest breathing, and bending movements of finger, elbow, and knee joints, and could reliably distinguish these different behavioral patterns.

## Data Availability

Data are contained within the article.
